# Protocol, rationale and design of PEOPLE (Post ExpOsure Prophylaxis for LEprosy in the Comoros and Madagascar): a cluster randomized trial on effectiveness of different modalities of implementation of post-exposure prophylaxis of leprosy contacts

**DOI:** 10.1186/s12879-019-4649-0

**Published:** 2019-12-05

**Authors:** Nimer Ortuno-Gutierrez, Assoumani Younoussa, Andriamira Randrianantoandro, Sofie Braet, Bertrand Cauchoix, Stéphanie Ramboarina, Abdallah Baco, Aboubacar Mzembaba, Zahara Salim, Mohamed Amidy, Saverio Grillone, Jan Hendrik Richardus, Bouke C. de Jong, Epco Hasker

**Affiliations:** 1 Projects Department, Damien Foundation, Boulevard Leopold II, 263, PO B-1081 Brussels, Belgium; 2 National Tuberculosis and Leprosy control Program, Moroni, Union of the Comoros; 3 National Leprosy control Programme, Antananarivo, Madagascar; 4 Institute of Tropical Medicine, Antwerp, Belgium; 5 Raoul Follereau, Antananarivo, Madagascar; 6 Erasmus MC, University Medical Center Rotterdam, Rotterdam, The Netherlands

**Keywords:** Single dose rifampicin, Mapping, Clustering, Post exposure prophylaxis, Acceptability, Cost-effectiveness

## Abstract

**Background:**

Leprosy is an ancient infectious disease with a global annual incidence that has plateaued above 200,000 new cases since over a decade. New strategies are required to overcome this stalemate. Post-exposure prophylaxis (PEP) with a single dose of Rifampicin (SDR) has conditionally been recommended by the World Health Organization (WHO), based on a randomized-controlled-trial in Bangladesh. More evidence is required. The Post ExpOsure Prophylaxis for Leprosy (PEOPLE) trial will assess effectiveness of different modalities of PEP on the Comoros and Madagascar.

**Methods:**

PEOPLE is a cluster-randomized trial with villages selected on previous leprosy-incidence and randomly allocated to four arms. Four annual door-to-door surveys will be performed in all arms. All consenting permanent residents will be screened for leprosy. Leprosy patients will be treated according to international guidelines and eligible contacts will be provided with SDR-PEP.

Arm-1 is the comparator in which no PEP will be provided. In arms 2, 3 and 4, SDR-PEP will be provided at double the regular dose (20 mg/kg) to eligible contacts aged two years and above. In arm 2 all household-members of incident leprosy patients are eligible. In arm 3 not only household-members but also neighbourhood contacts living within 100-m of an incident case are eligible. In arm 4 such neighbourhood contacts are only eligible if they test positive to anti-PGL-I, a serological marker. Incidence rate ratios calculated between the comparator arm 1 and each of the intervention arms will constitute the primary outcome.

**Discussion:**

Different trials on PEP have yielded varying results. The pivotal COLEP trial in Bangladesh showed a 57% reduction in incidence over a two-year period post-intervention without any rebound in the following years. A study in a high-incidence setting in Indonesia showed no effect of PEP provided to close contacts but a major effect of PEP provided as a blanket measure to an entire island population. High background incidence could be the reason of the lack of effect of PEP provided to individual contacts. The PEOPLE trial will assess effectiveness of PEP in a high incidence setting and will compare three different approaches, to identify who benefits most from PEP.

**Trial registration:**

Clinicaltrials.Gov. NCT03662022. Initial Protocol Version 1.2, 27-Aug-2018.

## Background

We describe the protocol of the PEOPLE study, a randomized controlled trial on post exposure prophylaxis (PEP) for leprosy on the Comoros and Madagascar. Leprosy is an ancient infectious disease caused by *Mycobacterium leprae.* In humans, it is probably transmitted through the air provoking skin and nerve lesions after years without clinical manifestations. Delayed treatment leads to complications including permanent deformity, which in its turn leads to stigma.

Since 2000, leprosy has been declared eliminated as public health problem worldwide, on the basis of a prevalence rate of less than one per 10,000 population [1]. Leprosy incidence however has plateaued above 200,000 cases annually illustrating uninterrupted transmission. The study countries, Comoros and Madagascar, both have high leprosy incidence and are included in the list of 23 priority countries for leprosy control drawn up by the World Health Organization (WHO) [2]. The islands of Anjouan and Mohéli on the Comoros have been reporting incidence rates close to 10 per 10,000 population for years. In some villages on Anjouan door-to-door screening in 2017 revealed prevalence rates of up to 2% [3]. Madagascar notified 1424 new leprosy cases in 2018 on a population of approximately 26 million, 9% were children. (2) However, the epidemiological burden varies between the districts, explained by different access related issues such as geographical, availability of qualified health staff, health-seeking awareness, etc. For this study, a hyperendemic district, Miandrivazo, has been selected.

Providing a single dose of Rifampicin (SDR) as PEP to contacts of leprosy patients has been conditionally endorsed by WHO as a strategy to overcome the current stalemate [4, 5]. This recommendation is mainly based on the ‘contact transmission and chemoprophylaxis in leprosy’ (COLEP) trial in Bangladesh that demonstrated a 57% reduction of leprosy incidence over a two-year period following provision of SDR to household and social contacts of leprosy patients [6]. However in high endemicity settings in Indonesia, two monthly doses of Rifampicin administered to household and social contacts of leprosy patients had no effect, in contrast with providing PEP to an entire island population that resulted in a threefold reduction of leprosy incidence [7].

## Methods/design

### Objectives and hypothesis

In this study we intend to compare effectiveness as well as cost effectiveness of three different modalities of SDR-PEP to a comparator arm in which no PEP is provided.

### Study design

The study has been designed as a cluster randomized trial in which villages will be randomly allocated to four arms. All villages will be subject to four annual rounds of door-to-door screening. Leprosy patients identified will be treated in accordance with international guidelines, contacts will be provided PEP in accordance with the study arm. In arm 1, the comparator arm, no PEP will be provided. In arm 2 all asymptomatic household members will receive SDR-PEP. In arm 3 SDR-PEP will be provided to all leprosy asymptomatic household-embers plus neighbourhood contacts residing within a 100-m radius from an index case household. Finally, in arm 4 SDR-PEP will be provided to all household members and to those residing within 100-m of an index case and testing positive to anti-phenolic glycolipid-I (anti-PGL-I), a test for detection of IgM antibodies to *M. leprae*. If the village population in a 100-m radius around households of index cases comprises ≥50% in arm 3 or ≥ 75% in arm 4, the entire village will in principle be eligible for SDR-PEP.

### Setting

The Union des Comores is an island nation in the Indian Ocean, north of Madagascar. On the main island, Grande Comore, leprosy has become a rare disease but the islands of Anjouan and Mohéli are still notifying around 400 new leprosy cases annually on an estimated population of 450,000.

The Comoros has for decades had a strong leprosy control program, achieving good coverage and fully in line with the strategies recommended by WHO. Even though early case finding is achieved, with less than 3% of visible deformities in new leprosy patients, transmission has remained high. This is reflected in a 27% proportion of children under 15 years of age among new patients.

Madagascar has a population of 26 million and notifies around 1500 leprosy cases annually. However, leprosy control program coverage is patchy and case detection is often late, reflected in a proportion of new patients presenting with visible deformities close to 20%. The proportion of children among incident leprosy patients is lower than on the Comoros but remains significant at 9% [2]. The district of Miandrivazo selected for this study is located in the Menabe area on the central west of Madagascar. More specifically, the study takes place in the southern part of the district which is mainly rural and relatively sparsely populated with numerous small and remote villages. Coverage of leprosy control services in that area has been limited and reliable incidence data have not yet been available for recent years. The first round of screening in Madagascar will therefore be used as a baseline survey, randomization will only be done upon its completion.

### Participants

Participants will be recruited from 48 villages on the Comoros (32 on Anjouan and 16 on Mohéli) and a number of villages yet to be determined in Miandrivazo district of Madagascar. Leprosy screening will be offered to both genders and all ages, if required treatment will be provided. PEP however will only be provided to those permanent residents aged two years or above who did not receive Rifampicin in the past two years. Another exclusion criterion is having cough of more than two weeks’ duration (presumptive pulmonary tuberculosis).

### Randomization

On Anjouan and Mohéli (Comoros) the randomization has been done at village level based on reported leprosy incidence in the years 2013–2017. Villages had been grouped by island in decreasing order of incidence in blocks of four. Within each block villages were randomly allocated to one of the four study arms in a mutually exclusive manner, using random numbers generated in Excel.

For Miandrivazo district (Madagascar) the randomization will be done at the end of the first year of 2019 after completion of the door-to-door active case detection carried out during the first year of the study. A number of high prevalence villages with a total population of ideally close to 20,000 will be selected, taking care of having a fourfold (e.g. 16 or 20). These will then be grouped into blocks of four based on prevalence and randomized over the four study arms within each block similarly to the procedure used on the Comoros.

### Outcome measures

The principal outcome measure will be the leprosy incidence rates in each of the four study arms. The incidence rates in the Comoros will be measured between the first and the fourth door-to-door survey, while the incidence rates in Madagascar will be evaluated between the second and fourth survey round. The incidence rate ratios will then be calculated between the comparator arm (arm 1) and each of the intervention arms. In addition, the costs of screening and PEP provision in each of the four arms will be determined allowing to calculate a cost per leprosy case averted for each of the intervention arms with the comparator arm as baseline. Spatial clustering of leprosy at sub-village level will be assessed by comparing incidence rates within households of index cases and incidence among neighbourhood contacts at varying distances (< 25 m, 25–50 m, 50–75 m and 75–100 m) to incidence rates among those living at more than 100 m from any index case.

### Intervention implementation and data collection

Door-to-door screening will be conducted, covering all study villages once yearly for a total of four consecutive years. In addition to leprosy the study will focus on skin diseases. Treatment will be provided for common minor skin conditions such as fungal infections or scabies. Leprosy patients detected will be treated according to the guidelines from the national leprosy control program.

In each household screened, name, age and gender of each permanent household member will be recorded on a paper form during the visit. This form, of which one copy will be used per household, has a unique serial number. It has one line per person, each line with a space to sign for informed consent and a pre-printed unique barcode. These forms will be used to enter form serial number and name, age, gender and barcode of each individual in a database in MS Access. All other data will be recorded through an Android application made in Open Data Kit Collect (ODK). The serial number will be copied from the paper form into the app for each household, for each individual the corresponding barcode will be scanned. Apart from form serial number and barcodes, the app will also be used to record the date of visit, Global Positioning System (GPS) coordinates of the household, village name, and number of household members. For each household member we will record whether the person was present, whether the person has a history of leprosy, whether (s)he was examined and what was the result of the clinical exam. We will also record the presence or absence of a Bacille de Calmette Guérin (BCG) scar and ask for cough of more than two week’s duration. These data are uploaded to a secure server whenever a village is completed. Names will not be recorded in the Android application but can be retrieved from the Access database if required based on the barcodes (e.g. for treatment of leprosy). Thus, exact records will be available on numbers of persons living in the households visited, numbers screened, numbers of cases identified and the date and location of screening activities.

Participants with cough for more than two weeks identified during surveys will have a sputum sample collected for tuberculosis screening. Those with confirmed tuberculosis will be treated according to the national tuberculosis guidelines.

Leprosy diagnosis will be clinical, based on the presence of three cardinal signs: patch with loss of sensation, enlarged peripheral nerves and/or slit-skin smear (SSS) positive for acid fast bacilli. All leprosy cases diagnosed will be verified by experienced leprosy national control program staff. If confirmed they will be treated according to WHO guidelines. Conditional upon their informed consent, incident leprosy patients will be enrolled in a sub study in which slit skin smears, nasal swabs and skin biopsies will be sampled. Biopsies will be subjected to quantitative polymerase chain reaction (qPCR) for *Mycobacterium leprae*. In the framework of a sub-study, not part of this protocol, genotyping of bacillary DNA will be performed on all qPCR positive samples.

SDR-PEP will be provided to all household members in arms 2, 3 and 4 as soon as a new leprosy case is detected. Children below two years of age and persons having received Rifampicin within the last 24 months will be excluded. In Comoros, a new leprosy case is defined in the first round as a case arising after the 31st of December, 2017 (or after the 31st of December, 2018 in Madagascar), and in subsequent rounds as a case arising after the previous screening round.

In arms 3 and 4 PEP will also be provided to neighbourhood contacts living within 100 m of an index case. This will only be done once the entire village has been screened. Selection of the group of individuals living within 100 m of an incident case will be done after analysing cleaned data by the principal investigators in each island and the research coordinator at the Institute of Tropical Medicine, Antwerp. As explained earlier, in arm 4 non-household contacts living within 100 m of an index case are eligible for PEP only if they are positive to anti-PGL-I. In arm 3, if 50% or more of the population live within 100 m of an index case, the entire village will be considered eligible. This will be the case in arm 4 if 75% or more live within 100 m of an index case.

Detailed costs of screening and PEP implementation will be recorded for each study arm including direct costs for direct implementation, monitoring and support.

### Post exposure prophylaxis

As post exposure prophylaxis we will use a single dose of Rifampicin, in accordance with the WHO guidelines. However, the dose used will be higher than the standard dose. Rifampicin has for decades been a core drug for the treatment of tuberculosis (TB). The dosage of 10 mg/kg recommended in the 1970s was established balancing concerns on toxicity and cost [8] However, this dose might not be optimal in terms of efficacy. A study using Rifampicin at 20 mg/kg daily in treatment of TB demonstrated a doubling in early bactericidal activity compared to the standard dose [9] In another TB study, two weeks of Rifampicin at a dose of 35 mg/kg was well tolerated without increase in toxicity [8] The recent study ‘Optimization of the TB Treatment Regimen Cascade (OneRIF, ClinicalTrials.gov Identifier: NCT02153528)’ documented no increased toxicity in 475 adults treated with Rifampicin at 20 mg/kg for six months as part of the treatment of drug-susceptible TB, compared to 468 adults that were treated with Rifampicin at 10 mg/kg.

There are also precedents of using Rifampicin at high doses for leprosy post-exposure prophylaxis and treatment. In the French Polynesia Rifampicin at 25 mg/kg was used as post-exposure prophylaxis [10–12]. Single dose of Rifampicin at 40 mg/kg was effective and safe for the treatment of PB cases with a negative bacillary index [13]. In the PEOPLE trial we therefore opted for Rifampicin in a single a dosage of 20 mg/kg, which we will refer to as ‘Single Double Dose Rifampicin Post Exposure Prophylaxis’ or ‘SDDR-PEP’.

SDDR-PEP will be provided under supervision of a village health worker who will keep a record of each person eligible and whether or not this person has taken his dose. As higher Rifampicin dosage has been documented safe [8, 10–12], we will implement passive adverse events (AE) surveillance. An AE is defined as any unexpected event in a clinical investigation after administering a pharmaceutical product, it does not necessarily imply a causal relationship. A distinction will be made between AE’s and serious AE’s (SAE), the latter defined as an AE that provokes death or is life-threatening, requiring hospitalization or increase in duration of existing hospitalization, or results in permanent disability /incapacity or provokes a congenital anomaly/ birth defect. We will record all AE and SAE occurring within 72 h of Rifampicin administration and classify them according to severity and probable relationship to SDDR-PEP. Also, health workers in charge of the selected villages will be informed about the PEOPLE trial and advised to report any AE or SAE. In case of SAE a specific template will be recorded and sent to pharmacovigilance unit of ITM within 24 h.

It has been documented that the risk of inducing Rifampicin resistance in undiagnosed TB or leprosy as a result of a single dose of Rifampicin is negligible [14]. Rifampicin resistance in TB can occur under monotherapy but that requires longer exposure [15]. In order to minimize the risk of Rifampicin resistance we will screen for TB and exclude all presumptive TB with cough of more than two weeks. Also, every person that has received Rifampicin within less than two years will be excluded from SDR-PEP. Finally, we will monitor Rifampicin resistance in leprosy through molecular testing on qPCR positive samples from leprosy cases.

Rifampicin interacts with drugs such as anti-retrovirals (ARV) [16] that are known as inducer of a number of genes controlling drug metabolism and transport like cytochrome P450 isoenzymes and the drug efflux pump p-glycoprotein. Therefore, concentration of Rifampicin administered with ARV may decrease. As we are providing a single dose and given that the Rifampicin serum half-life is less than five hours such interaction effect can be considered negligible irrespective of the dosing [17].

### Rationale

Effectiveness of PEP probably depends on the leprosy epidemiological burden, the type of contacts targeted and the type of PEP-regimen. Targeting household contacts only avoids issues of confidentiality but may lack effectiveness. Also targeting social contacts, as was done in the COLEP trial in Bangladesh, is probably more effective [6]. However, this approach may even not be effective in hyper endemic settings as suggested in a study performed in Indonesia [18].

Another important factor to consider is the regimen used. In the COLEP trial a 50–60% reduction in incidence was achieved after administering a single dose of Rifampicin at 10 mg/kg [6]. An expert committee convened in preparation of the PEP++ trial, which is to start soon, drafted a reinforced PEP regimen based on three monthly doses of Rifampicin plus Clarithromycin [19]. In the PEOPLE trial we choose to adopt a regimen that includes only one single dose of PEP for logistical and cost reasons.

### Data analysis

For our main analysis we will fit a random effects Poisson model (or negative binomial if overdispersed) comparing incidence rates between the first and fourth survey round in arms 2, 3 and 4 with those of the comparator arm 1 (starting from the second survey round in Madagascar). As random effects we will use island (Anjouan, Mohéli or Madagascar), ‘block’, i.e. the groups of four villages arranged in order of incidence initially used in the randomization process and village. We will thus obtain incidence rate ratios between arm 1 and each of the other arms. Considering the fact that three comparisons will be performed, a *p*-value of 0.017 will be used as threshold of statistical significance.

Spatial clustering will be assessed by calculating for each individual the distance to the nearest leprosy affected household in the previous year [20]. Household coordinates will be plotted in Quantum GIS version 3.8 Zanzibar. We will then calculate the distance to the nearest index case household for each household using the distance matrix tool. As a next step subjects will be divided into six categories: household contacts, neighbours at less than 25 m and neighbourhood contacts between 25 and 50 m, 50–75 m and 75–100 m and at more than 100 m. Incidence rate ratios for leprosy will be calculated with individuals living at more than 100 m as reference category and village as random effect.

Results of spatial analysis will be triangulated with results of phylogenetic of *M. leprae* clustering observed in the sub study in which skin biopsy samples are collected from each consenting incident leprosy patient.

Both average cost per person screened for leprosy per island and the average cost per leprosy patient detected per study arm will be assessed. Cost data will be gathered throughout the study. All incremental costs will be calculated by using comparator arm as a baseline.

### Sample size

The calculation of sample size is based on the primary objective and according to the methodology described by Hayes and Bennet for pair matched randomized controlled trials [21]. The incidence rate in the comparator arm with no PEP will be compared to each of the three intervention arms. We assumed that the annual incidence rate in the comparator arm will be 1.5/1000, based on data from 48 villages on the Comoros for the years 2013 to 2017. We expected a reduction of the leprosy incidence of 50% in any of the intervention arms. As three comparisons will be made, we opted for a significance level of 0.017.

Based on data from the Comoros we calculated a coefficient of variation k between clusters of 0.29. With an average cluster size of 2400, to achieve a power of 80%, we will need 13 clusters per study arm, i.e. 4* 31,200 participants. In order to compensate for inaccuracies in census data, as well as for absentees and non-responders, we decided to aim for 36,000 participants per study arm, i.e. 15% extra. Therefore, the total sample size is expected to be 124,000 and 20,000 to be recruited in the Comoros and in Madagascar respectively.

### Ethics

The study will be carried out according to the principles stated in the Declaration of Helsinki, all applicable regulations and according to established international scientific standards. The choice of treatment for the leprosy patients will not depend on the results of the PEOPLE study, but on the current national leprosy guidelines.

The study has been approved by the ‘Comité d’Éthique de la Recherche Biomédicale’ (CERBM) in Madagascar and the ‘Comité National d’Éthique pour les Sciences de la Vie et de la Santé’ (CNESS) in the Comoros. Approval has also been obtained from the Institutional Review Board (IRB) of ITM given that ITM is the sponsor of this study. In addition, the study has been approved by the Ethics Committee (EC) of the University of Antwerp Hospital in Antwerp.

Prior to the start, this study has been included in the Clinicaltrials.gov public registry (NCT03662022, on 7 September 2018, https://clinicaltrials.gov/ct2/show/NCT03662022).

## Discussion

Although single dose Rifampicin post exposure prophylaxis has now been conditionally endorsed by WHO [5], a lot of debate remains. Some argue that SDR will mainly prevent paucibacillary leprosy which is less infectious or that SDR may just postpone new cases rather than preventing them. Others argue that SDR might eventually lead to resistance of *M. leprae* against Rifampicin [22]. The pivotal COLEP trial in Bangladesh did not provide any such indications. There was a clear incidence reduction of 50–60%, without any rebound after the intervention ended [6]. However, evidence from more than one site is required and other options of PEP need to be explored. As was explained earlier we do opt for a higher dose of Rifampicin, hoping the increased early bactericidal effect observed for *M. tuberculosis* will also apply to *M. leprae*.

The PEOPLE trial is the second major randomized controlled trial on post-exposure prophylaxis for leprosy after the COLEP trial. It is implemented against a background of very high leprosy incidence, which may have a major implication for effectiveness of PEP. The PEOPLE trial therefore includes two different approaches to PEP, targeting only household members and targeting entire communities. In addition, we will assess the feasibility and effectiveness of selecting neighbourhood contacts eligible for PEP based on presence of antibodies against *M. leprae*.

Use of innovative digital tools for data collection and mapping allows geospatial patterns in leprosy transmission to be assessed, which can at a later stage be triangulated with results of phylogenetic clustering genotypes of *M. leprae* found in the patients. PCR results from the sub study will also allow the accuracy of diagnostic procedures to be validated.

Costing and cost effectiveness are also part of the study. Thus, once completed we will be able to provide answers relevant for leprosy control programs concerned with questions on feasibility and cost of PEP implementation.

As a by-product of this study we are establishing a very well characterized cohort of leprosy patients for which clinical information, samples and precise geographical location will be available. This cohort would allow other pertinent research questions to be answered such as risks for relapse and drug resistance [23].

## Data Availability

The data supporting the findings of this publication will be retained at the Institute of Tropical Medicine, Antwerp and will not be made openly accessible due to ethical and privacy concerns. Data can however be made available after approval of a motivated and written request to the Institute of Tropical Medicine at ITMresearchdataaccess@itg.be.

## Abbreviations

AE: Adverse Event
Anti-PGL-I: Anti-phenolic glycolipid-I
AR: Adverse Reaction
BCG: Bacille de Calmette Guérin
DF: Damien Foundation
EDCTP: European and Developing Countries Clinical Trial Partnership
FRF: Fondation Raoul Follereau
IRB: Institutional Review Board
ITM: Institute of Tropical Medicine
MB: Multi bacillary
NTLCP: National Tuberculosis and Leprosy Control Program
ODK: Open Data Kit Collect
PEOPLE: Post ExpOsure Prophylaxis for LEprosy in the Comoros and Madagascar
PEP: Post Exposure Prophylaxis
qPCR: Quantitative Polymerase Chain Reaction
SAE: Serious Adverse Event
SDDR-PEP: Single Double Dose Rifampicin Post-Exposure Prophylaxis
SDR: Single Dose Rifampicin
SSS: Slit-skin smears

## References

[1] World Health Assembly. World Health Assembly 44. Resolutions and decisions. Leprosy: World Health Organization 1991 [Available from: http://www.who.int/neglected_diseases/mediacentre/WHA_44.9_Eng.pdf?ua=1. Accessed Aug 2019.

[2] World Health Organization. Weekly epidemiological record. Global leprosy update, 2018: moving towards a leprosy-free world Geneva: World Health Organization; 2019 [Available from: https://www.who.int/wer/2019/wer9435_36/en/. Accessed Aug 2019.

[3] Ortuno-Gutierrez N, Baco A, Braet S, Younoussa A, Mzembaba A, Salim Z (2019). Clustering of leprosy beyond the household level in a highly endemic setting on the Comoros, an observational study. BMC Infect Dis.

[4] World Health Organization. Global Leprosy Strategy 2016 - 2020. Accelerating towards a leprosy-free world New Delhi: WHO Library Catologuing-in-Publication data; 2016 [Available from: https://apps.who.int/iris/bitstream/handle/10665/208824/9789290225096_en.pdf?sequence=14&isAllowed=y. Accessed Aug 2019.

[5] World Health Organization. Guidelines for the Diagnosis, Treatment and Prevention of Leprosy New Delhi: Regional Office for South-East Asia; 2018 [Available from: https://apps.who.int/iris/bitstream/handle/10665/274127/9789290226383-eng.pdf?ua=1. Accessed Aug 2019.

[6] Moet FJ, Pahan D, Oskam L, Richardus JH (2008). Effectiveness of single dose rifampicin in preventing leprosy in close contacts of patients with newly diagnosed leprosy: cluster randomised controlled trial. BMJ..

[7] Bakker MI, Hatta M, Kwenang A, Van Benthem BH, Van Beers SM, Klatser PR (2005). Prevention of leprosy using rifampicin as chemoprophylaxis. Am J Trop Med Hyg..

[8] Boeree MJ, Diacon AH, Dawson R, Narunsky K, du Bois J, Venter A (2015). A dose-ranging trial to optimize the dose of rifampin in the treatment of tuberculosis. Am J Respir Crit Care Med.

[9] Diacon AH, Patientia RF, Venter A, van Helden PD, Smith PJ, McIlleron H (2007). Early bactericidal activity of high-dose rifampin in patients with pulmonary tuberculosis evidenced by positive sputum smears. Antimicrob Agents Chemother.

[10] Cartel JL, Chanteau S, Boutin JP, Taylor R, Plichart R, Roux J (1989). Implementation of chemoprophylaxis of leprosy in the southern Marquesas with a single dose of 25 mg per kg rifampin. Int J Lepr Other Mycobact Dis.

[11] Nguyen LN, Cartel JL, Grosset JH. Chemoprophylaxis of leprosy in the southern Marquesas with a single 25 mg/kg dose of rifampicin. Results after 10 years. Lepr Rev. 2000;71 Suppl:S33–5; discussion S5–S6. 10.5935/0305-7518.20000064 11201884

[12] Wendt WR (1978). Recuperation cure for schoolchildren indication and own experience (author's transl). Offentl Gesundheitswes.

[13] Pattyn SR, Groenen G, Janssens L, Kuykens L, Mputu LB (1991). A controlled therapeutic trial in paucibacillary leprosy comparing a single dose of rifampicin with a single dose of rifampicin followed by one year of daily dapsone. The collaborative study Group for the Treatment of leprosy in Zaire. Lepr Rev.

[14] Mieras L, Anthony R, van Brakel W, Bratschi MW, van den Broek J, Cambau E (2016). Negligible risk of inducing resistance in Mycobacterium tuberculosis with single-dose rifampicin as post-exposure prophylaxis for leprosy. Infect Dis Poverty.

[15] Mitchison DA (1998). How drug resistance emerges as a result of poor compliance during short course chemotherapy for tuberculosis [counterpoint]. Int J Tuberc Lung Dis.

[16] Cohen K, Meintjes G (2010). Management of individuals requiring antiretroviral therapy and TB treatment. Curr Opin HIV AIDS.

[17] Donald PR, Maritz JS, Diacon AH (2011). The pharmacokinetics and pharmacodynamics of rifampicin in adults and children in relation to the dosage recommended for children. Tuberc..

[18] Bakker MI, Hatta M, Kwenang A, Van Benthem BHB, Van Beers SM, Klatser PR (2005). Prevention of leprosy using rifampicin as chemoprophylaxis. Am J Trop Med Hyg..

[19] Mieras LF, Taal AT, van Brakel WH, Cambau E, Saunderson PR, Smith WCS (2018). An enhanced regimen as post-exposure chemoprophylaxis for leprosy: PEP. BMC Infect Dis.

[20] Rezaeian M, Dunn G, St Leger S, Appleby L (2007). Geographical epidemiology, spatial analysis and geographical information systems: a multidisciplinary glossary. J Epidemiol Community Health.

[21] Hayes RJ, Bennett S (1999). Simple sample size calculation for cluster-randomized trials. Int J Epidemiol.

[22] Lockwood DNJ, Krishnamurthy P, Kumar B, Penna G (2018). Single-dose rifampicin chemoprophylaxis protects those who need it least and is not a cost-effective intervention. PLoS Negl Trop Dis.

[23] Balagon MF, Cellona RV, Cruz E, Burgos JA, Abalos RM, Walsh GP (2009). Long-term relapse risk of multibacillary leprosy after completion of 2 years of multiple drug therapy (WHO-MDT) in Cebu, Philippines. Am J Trop Med Hyg.

